# The prevalence of hepatitis C virus infection in *β*-thalassemia patients in Pakistan: a systematic review and meta-analysis

**DOI:** 10.1186/s12889-020-8414-5

**Published:** 2020-04-29

**Authors:** Sohail Akhtar, Jamal Abdul Nasir, Andrew Hinde

**Affiliations:** 1 Department of Statistics, Government College University, Lahore, Katchery Road, Lahore, Pakistan; 2 Southampton Statistical Sciences Research Institute, University of Southampton, Southampton, UK

**Keywords:** Prevalence, HCV, *β*-Thalassemia, Pakistan, Systematic review, Meta-analysis

## Abstract

**Background:**

Hepatitis C virus infection is the most commonly reported bloodborne infection in Pakistan. Frequent blood transfusions in *β*-thalassemia patients expose them to a high risk of HCV infection. The purpose of this paper is to summarise the current data on the prevalence of HCV infection in *β*-thalassemia patients in Pakistan by using a systematic review and meta–analysis.

**Methods:**

PubMed, EMBASE, Web of Sciences, the Cochrane Library, Directory of Open Access Journal and local databases were systematically searched for studies published between January 1st, 1995 and May 31st, 2019. Meta-analysis was performed using the DerSimonian and Laird random-effects models with inverse variance weighting. The presence of publication bias was tested by Egger test, and the methodological quality of each included article was evaluated by the STROBE.

**Results:**

We identified a total of 229 potential studies, of which 27 studies were finally considered in the meta-analysis. The pooled prevalence of HCV in *β*-thalassemia patients in Pakistan was 36.21% (95% CI: 28.98–43.75%) based on 5789 *β*-thalassemia patients, but there was considerable heterogeneity. Meta-analysis estimated the HCV prevalence among the *β*-thalassemia patients at 45.98% (95% CI: 38.15–53.90%) in Punjab, 31.81% (95% CI: 20.27–44.59%) in Sindh, and 28.04% (95% CI: 13.58–45.26%) in Khyber Pakhtunkhwa. Meta–regression analysis showed that geographical location was a key source of heterogeneity.

**Conclusions:**

The pooled prevalence of HCV in *β*-thalassemia patients in Pakistan was more than one in three, and higher than in neighbouring countries. It varies regionally within the country. With the use of standard prevention procedures during blood transfusion, the risk of HCV transmission in *β*-thalassemia patients could be controlled and the prevalence of HCV in *β*-thalassemia patients reduced.

## Background

The *β*-thalassemias are among the most common genetic diseases and affect millions of children throughout the world [1]. Around 1.5% (80–90 million people) of the worldwide population are carriers for *β*-thalassemia, with 50,000–60,000 new *β*-thalassemia cases being born each year [2]. *β*-thalassemia is most prevalent in the populations of Asia, the Indian subcontinent, the Mediterranean countries, Africa and the Middle East [3–5]. In Pakistan, *β*-thalassemia is one of the commonest inherited disorders, with a carrier frequency of 5 to 7% of the Pakistani population [2]. *β*-thalassemia patients are now surviving to older ages due to the availability of blood transfusion and iron chelation. There are around 100,000 patients registered currently but the burden of disease is increasing, with 5000 to 9000 children born with the disorder annually [6].

Bloodborne infections are the second commonest reason of death in *β*-thalassemia patients in Pakistan [2]. Regular blood transfusions in *β*-thalassemia patients expose them to a higher risk of contracting HCV viral infection, especially if adequate viral screening of blood donors has not been undertaken. The infection risk in β-thalassemia patients acts as a marker for the risk of transfusion-transmitted infections in the general population as their exposure to blood transfusions is high. If the infection rate is low in *β*-thalassemia patients it implies that the risk for the general population will be minimal.

Hepatitis C infection is one of the most common bloodborne infections. More than 10 million individuals are living with HCV infection in Pakistan, and hence vulnerable to high morbidity and mortality [7]. Pakistan is a developing country: according to the Human Development Index of the United Nations, it stands in 150th position out of 189 countries and territories [8]. The health standard in Pakistan is well below the international standards to which all countries aspire. Therefore, contaminated blood transfusion is still a main risk factor for the spread of HCV. This is due to the lack of screening and the widespread use of paid blood donors [9]. Several studies have been published on the prevalence of HCV in *β*-thalassemia patients in Pakistan and there is considerable variation in the prevalence reported in the individually published studies. The purpose of this study is to investigate the pooled prevalence based on the available published studies conducted on the prevalence of HCV infection in *β*-thalassemia patients, and to describe its associated risk factors in Pakistan. To our knowledge, this is the first systematic review and meta-analysis to investigate the pooled prevalence of HCV infection in *β*-thalassemia patients in the country.

## Methods

### Search strategy

A systematic literature search on PubMed, EMBASE, the Cochrane Library, Web of Sciences, Directory of Open Access Journal and Pakistani Journals Online websites was conducted by two authors (J.A.N. and S.A.) to find studies performed on the prevalence of HCV infection in *β*-thalassemia patients and published from January 1st, 1995 to May 31st 2019. Using MeSH headings, we searched for, the terms “prevalence”, “epidemiology”, “seroprevalence”, “hepatitis C Virus”, “HCV”, “hepacivirus”, “hep C,” “thalassemia”,” *β*-thalassemia”, “thalassemia major”, “multitransfused blood transfusion”, “patients”, “Pakistan”, and “Pakistani”, as well as variations thereof. The results were defined using the Preferred Reporting Items for Systematic and Meta-analyses (PRISMA) statement (Table 1) [10], and the PRISMA 2009 checklist is attached in supplementary file S1.

**Table 1:** Description and list of characteristics of the included studies

Author	Year	Study Design	Sample size	Cases	Prevalence (%)	Setting	Province	Sex	Working Year	% Female	% Male	Average Age	Test	Quality
Bhatti et al.[18]	1995	NA	35	21	60.00	Urban	Khyber Pakhtunkhwa	Both	NA	14.28	85.71	6.5	ELISA	Medium
Muhammad et al. [19]	2003	Cross-sectional	80	29	36.25	Urban	Khyber Pakhtunkhwa	Both	Jul. 1999 to Mar. 2001	NA	NA	7.5	ELISA	Medium
Shah at al. [20]	2005	Cross-sectional	250	142	56.80	Urban	Khyber Pakhtunkhwa	both	Jan. 2000 to Jan. 2001	72.00	28	10	ELISA	Medium
Hussain [21]	2008	Cross-sectional	180	75	41.67	Urban	Khyber Pakhtunkhwa	Both	Jan. 2002 to Dec. 2003	NA	NA	6.8	ELISA	Good
Ali et al. [22]	2011	NA	167	26	15.57	Urban	Khyber Pakhtunkhwa	Both	NA	62.28	36.7	NA	RNA	Medium
Khattak et al.[23]	2013	NA	170	37	21.67	Urban	Khyber Pakhtunkhwa	both	Jan. 2012 to Dec. 2012	55.29	44.71	10	ELISA	Medium
Khan et al. [24]	2015	Cross-sectional	180	14	7.77	Urban	Khyber Pakhtunkhwa	Both	Jun. 2013 to Jul. 2014	38.89	61.11	NA	NA	Medium
Shah et al. [25]	2018	NA	324	18	5.56	Urban	Khyber Pakhtunkhwa	Both	Oct. 2013 to Mar. 2014	34.50	60.23	15.5	RNA	Medium
Younus et al. [26]	2004	Cross-sectional	75	32	42.00	Urban	Punjab	Both	Jul. to Sep. 2003	64.00	36	6.5	ELISA	Good
Iqbal at el. [27]	2010	NA	141	50	35.46	Urban	Punjab	Both	Sep. 2008 to Aug. 2009	58.20	41.8	8	ELISA	Medium
Ain et al. [28]	2011	Cross-sectional	300	195	65.00	Urban	Punjab	Both	NA	34.33	65.67	10	NA	Medium
Iqbal at el. [29]	2013	Cross-sectional	95	40	42.11	Urban	Punjab	Both	Oct. 2009 Apr. 2010	60.00	40	9.2	ELISA	Medium
Din et al. [30]	2014	NA	95	45	47.00	Both	Punjab	Both	Jul. 2017 to Sept. 2017	56.84	53.68	7	ELISA	Good
Nazir et al. [31]	2014	NA	200	82	41.00	Urban	Punjab	Both	Jan. 2013 to May 2013	12.00	88	8.5	ELISA	Medium
Saeed et al. [32]	2015	Cross-sectional	262	146	55.73	Urban	Punjab	Both	Nov. 2011 to Apr. 2012	40.07	59.92	9.26	ELISA	Medium
Sheikh et al. [33]	2015	Cross-sectional	145	99	68.27	Urban	Punjab	Both	Jan. 2009 to Dec. 2009	63.45	36.55	9	ELISA	Medium
Khan et al. [34]	2017	Cross-sectional	470	216	45.95	Urban	Punjab	Both	Mar. 2014 to Sep. 2014	65.96	34.04	4.8	ELISA	Medium
Rashid et al. [35]	2017	Cross-sectional	130	27	20.76	Urban	Punjab	Both	Jan. 2014 to Jun. 2014	60.00	40	9.7	ELISA	Medium
Raza et al. [36]	2018	Cross-sectional	200	82	41.00	Urban	Punjab	Both	Jan. 2015 to Dec. 2016	43.00	57	10.11	ELISA	Good
Mujeeb et al. [37]	1997	NA	91	46	50.54	Urban	Sindh	Both	NA	39.56	60.43	13	NA	Medium
Akhtar et al. [38]	2002	Cross-sectional	341	70	20.50	Urban	Sindh	Both	Jun-91	NA	NA	5	RNA	Good
Akhtar et al. [39]	2004	NA	86	38	44.20	Urban	Sindh	Both	NA	31.40	67.44	12	ELISA	Medium
Riaz et al. [40]	2011	Cross-sectional	79	34	43.00	Urban	Sindh	Both	Jul. 2009to Sep. 2009	41.77	58.23	12	ELISA	Medium
Ansari et al. [41]	2012	Cross-sectional	160	21	13.10	Urban	Sindh	Both	Jan. 2010 to Dec. 2010	49.38	50.63	8.5	ELISA	Medium
Sultan et al. [42]	2016	Cross-sectional	100	27	27.00	Urban	Sindh	Both	Jun. 2011 to Jun. 2014	54.00	46	15	ELISA	Good
Burki at el. [43]	2005	NA	180	75	41.67	Urban	Punjab + Khyber Pakhtunkhwa	Both	Jan. 2002 to Dec. 2003	NA	NA	6	ELISA	Medium
Kiani et al. [44]	2016	Cross-sectional	1253	273	21.71	Urban	Punjab + Sindh	Both	Jul. 2015 to Dec. 2015	46.21	53.79	10.1	NA	Medium

### Inclusion and exclusion criteria

Studies were included in this study if: (1) they were published in peer-reviewed journals; (2) they were conducted in Pakistan; (3) they reported on the prevalence HCV in thalassemia patients; (4) they were published in the English language.

Studies were excluded if: (1) they were in languages other than English; (2) they were case series, reviews, letters, and editorials or commentaries; (3) they did not allow the calculation of the prevalence of HCV; (4) they were duplicates (using the same data), in which case the more recently published version only was considered; (5) they related to the Pakistani community living outside Pakistan.

### Data extraction

After choosing the relevant articles, two reviewers (J.A.N. and S.A.) independently screened the titles and abstracts to identify articles for full-text read. The data was then extracted using a standardised data extraction template of Microsoft Office Excel 2013. Information extracted included: surname of first author, year of study, year of publication, geographic region (province), gender, study design, study setting (rural, urban or both), sample size and average age of β-thalassemia patients. Any disagreement regarding the extracted information was resolved by discussion and mutual consensus.

### Evaluating the quality of the included studies

Two authors (J.A.N. and S.A.) also independently judged the methodological quality of each included study using Quality Assessment Tool for Observational Cohort and Cross-Sectional Studies [11]. Any disagreement on the quality assessment check list was resolved by discussion and consensus. We categorized the quality of each included study as ‘good’ if its scored at least 70% of the points available, ‘medium’ if it scored 50–69%, and ‘poor’ if its scored less than 50%.

### Statistical analysis

Statistical analyses were conducted by the software R, version 3.5.3 [12], using two packages: ‘meta’ and ‘metafor’. Random effects (DerSimonian-Laird) models were used to make point estimates and their 95% confidence intervals (95% CIs), as well as to estimate the pooled prevalence of HCV among the *β*-thalassemia patients. A process for combining prevalence in the meta-analysis of multiple studies was used and the results presented in a forest plot. The Random effect models are more conservative than fixed effect models, and have better statistical properties in the presence of heterogeneity, as the random effects model allows both within-study and between-study variances [13, 14]. The Freeman–Tukey Double Arcsine transformation was used to stabilise the variance prior to the calculation of the pooled estimates [15]. Heterogeneity among the eligible articles was investigated with the *I*^2^ index [16]. For the *I*^2^-index, values of 75, 50, and 25% were considered high, moderate, and low levels of heterogeneity, respectively. To determine the possible reasons for substantial heterogeneity, univariate meta-regression and sub group analyses were conducted by geographical location, sample size, year of study, year of publication, gender and average age of the *β*-thalassemia patients. The presence of publication bias was evaluated by visually inspecting a funnel plot and test using Egger test [17], with *p*-value less than 0.05 indicating significant publication bias.

## Results

### Literature search

**Fig. 1:**
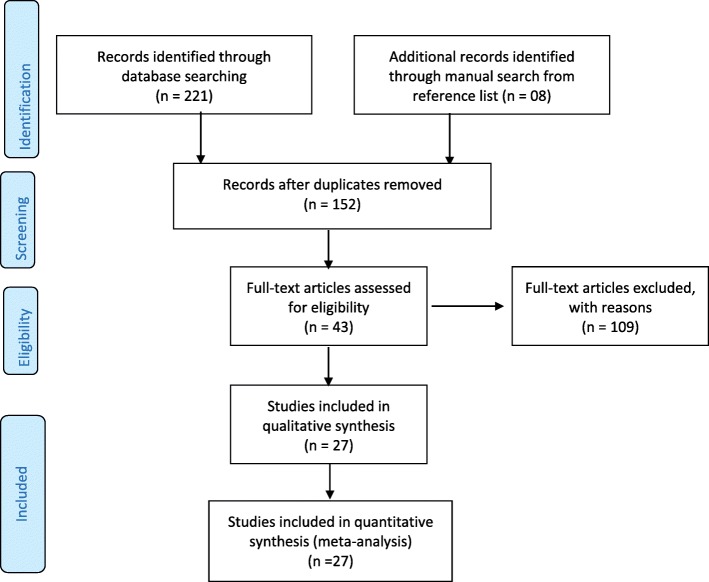
PRISMA 2009 flow diagram [10] explaining the number of included and excluded articles in the meta-analysis on the prevalence of HCV in *β*-thalassemia patients in Pakistan

Initially, 229 potential studies were identified. Of these, 77 were duplicates and were removed. We screened the titles and abstracts, and eliminated 109 irrelevant studies from the meta-analysis. As a result, only 43 articles were considered for full text review. Sixteen studies were eliminated after full text review: studies with no quantitative measures of hepatitis C virus in *β*-thalassemia patients; studies that were not conducted in Pakistan; studies that provided combined HCV and hepatitis B virus prevalence; studies based on duplicated data sets or that did not meet the eligibility criteria or that failed to include relevant indicators. In the end, 27 studies fulfilled the inclusion criteria and data were extracted. Figure 1 shows the study flow diagram (PRISMA) for the selection process [10].

### Characteristics of the included studies

The characteristics of the selected articles are summarised in Table 1. A cross-sectional research design was used in 17 studies, whereas ten studies did not clearly specify the research design. The articles were published between January 1995 and December 2018, while the period of participant inclusion was from June 1991 to September 2017. Three provinces of Pakistan were represented in the included studies: eight were conducted in Khyber Pakhtunkhwa [18–25], 11 were conducted in Punjab [26–36] and six were conducted in Sindh [37–42]. One study was conducted both in Punjab and Khyber Pakhtunkhwa [43] while one was conducted in Punjab and Sindh [44]. Most of the included studies (20 out of 27) reported (the) HCV prevalence based on the results of the ELISA (enzyme-linked immunosorbent assay) test [17–20, 22, 25, 26, 28–35, 40–43]. Only three studies reported the confirmation of HCV infection by RNA test [21, 24, 39]. Four studies did not explicitly mention the diagnostic methods theyused for HCV antibody reactivity testing [23, 27, 36, 38]. The sex of the patients was reported in 23 studies. The proportion of females ranged from 28.0 to 88.0%. The average age of patients varied from 4 years [34] to 15.5 years [25]. After reviewing the methodological quality of the studies, 6 were deemed to be of good quality, 21 of medium quality, and no article was found with poor quality. Sample size varied among studies with the smallest having a total of 35 patients [17] and the largest 1253 patients [35].

### Prevalence of HCV in β-thalassemia patients

**Fig. 2:**
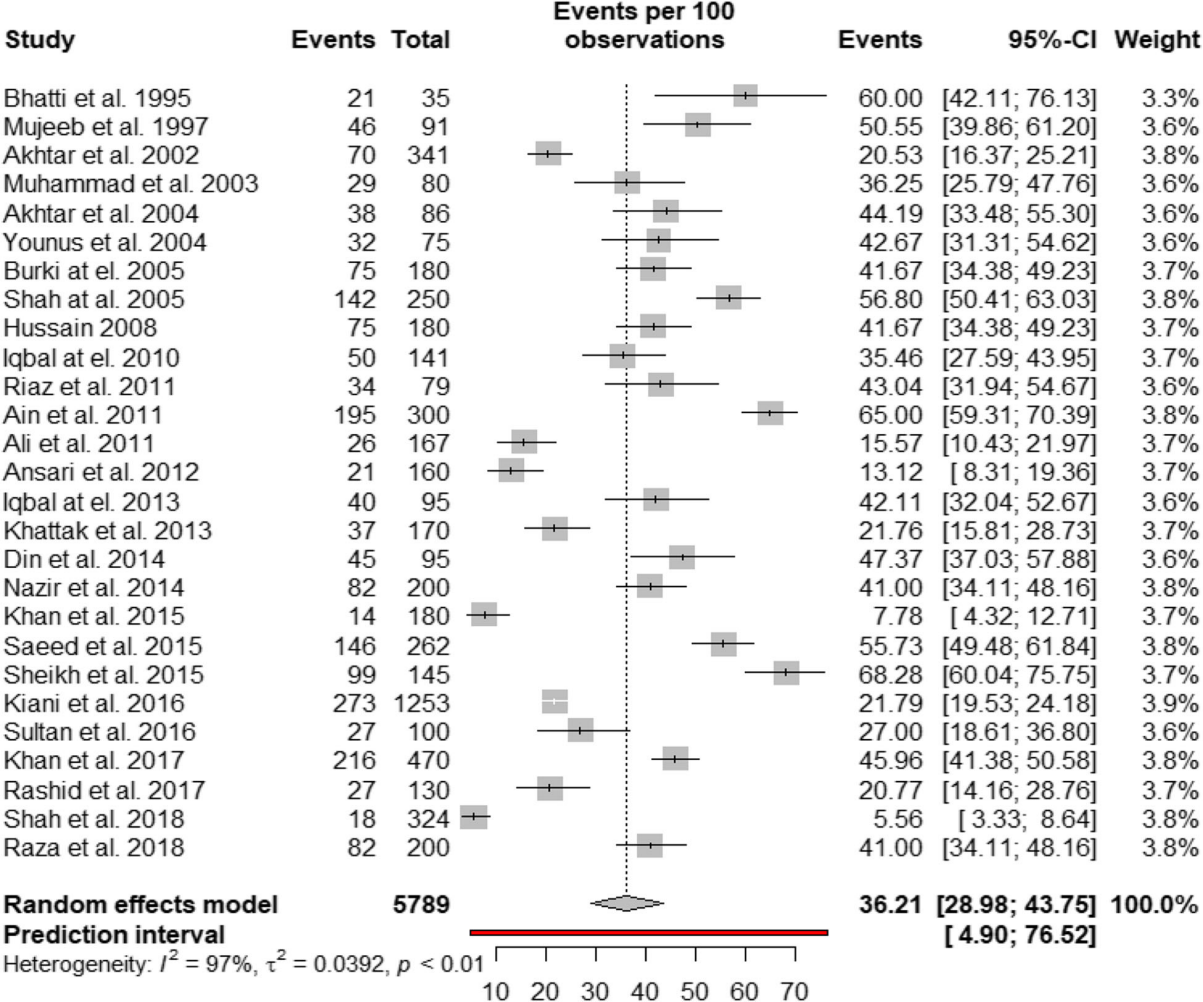
Forest plot of the prevalence of HCV infection among in *β*-thalassemia patients in Pakistan

**Fig. 3:**
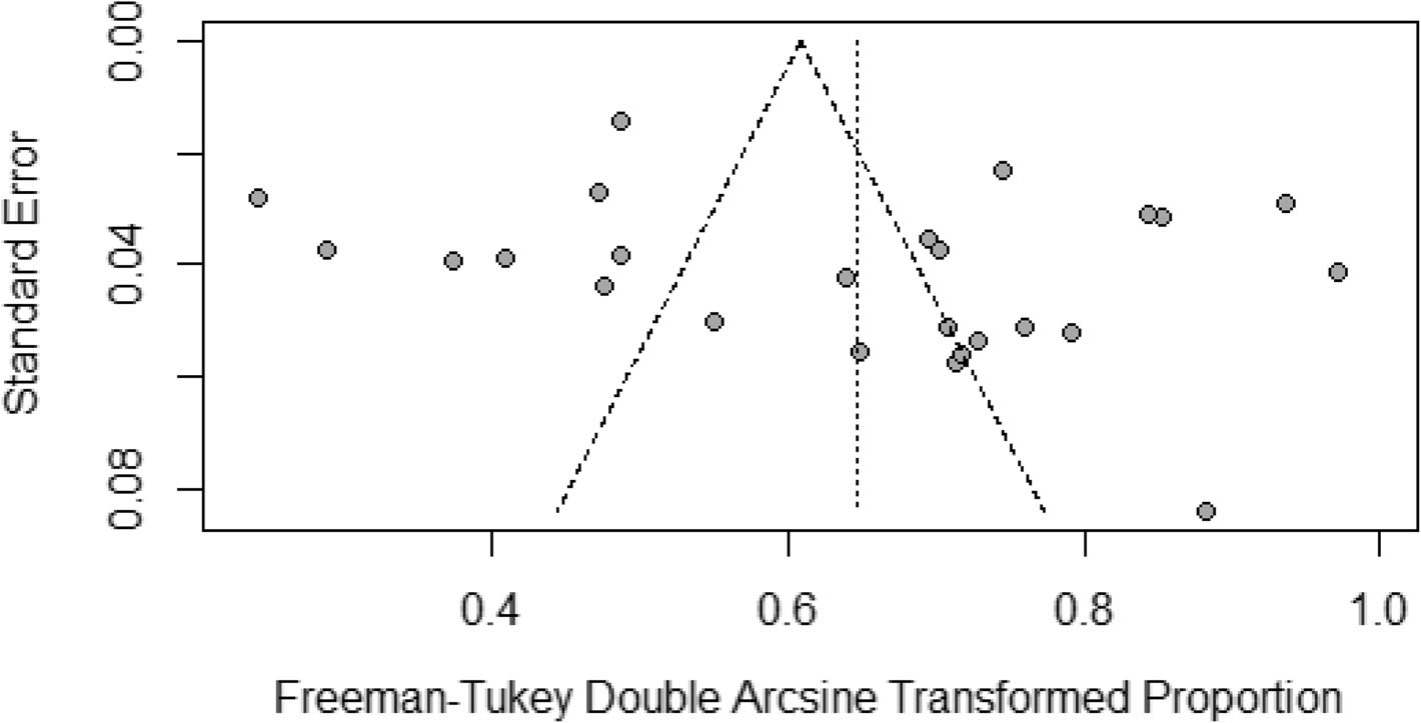
Funnel plot of the prevalence of HCV infection in *β*-thalassemia patients in Pakistan

Table 2 shows the summary of statistical analyses of the prevalence of the HCV in *β*-thalassemia patients in Pakistan. The overall prevalence of HCV infection in *β*-thalassemia patients was 36.21% (95% CI: 28.98–43.75%, *I*^2^ = 97.0%; 27 studies), based on a pooled sample of 5789. A forest plot of HCV prevalence in the *β*-thalassemia patients in the three provinces of Pakistan is presented in Fig. 2. Visual inspection of the funnel plot (Fig. 3) revealed some evidence of publication bias, but it was insignificant based on Egger test (*p* = 0.1506). Table 2 also presents the pooled prevalence of HCV in *β*-thalassemia patients for subgroups. The pooled subgroup prevalence stratified by geographical location (province) revealed that the prevalence of HCV in *β*-thalassemia patients was highest in Punjab at 45.98% (95% CI: 38.15–53.90%; *I*^2^ = 92.3%, based on 11 studies), compared with 31.81% (95% CI: 20.27–44.59%; *I*^2^ = 92.8%; based on 6 studies) in Sindh and 28.04% (95% CI: 13.58–45.26%, *I*^2^ = 97.6%; based on 8 studies) in Khyber Pakhtunkhwa. There was no significant difference in the prevalence of HCV between male (34.71% (95% CI: 23.32–47.04%)) and female (32.31% (95% CI: 20.17–45.75%)) *β*-thalassemia patients. The prevalence of HCV in *β*-thalassemia patients increased with age: the prevalence among those below 10 years of age was 33.87% (95% CI: 18.93–50.62%, *I*^2^ = 96.2%; 9 studies) while for those above 10 years it was 51.51% (95% CI: 34.52–68.34%, *I*^2^ = 96.2%; 9 studies). There was no publication bias in any subgroup.

**Table 2:** Summary statistics from meta-analyses of prevalence studies on HCV infection among *β*-thalassemia patients residing in Pakistan

	Studies	Sample	Cases	Prevalence % (95% confidence interval)	*I*^2^	Heterogeneity	*p* value for Egger’s test	*p* value for difference
Prevalence of HCV in *β*-thalassemia patients	27	5789	1960	36.2 (28.98–43.75)	0.970	< 0.001	0.1506	
**By sex**								0.7978
Male	12	1894	592	34.71 (23.32–47.04)	0.963	< 0.0001	0.2923	
Female	12	1316	364	32.31 (20.17–45.75)	0.952	< 0.0001	0.2304	
**By age**								0.1460
Less than 10 years	9	970	386	33.87 (18.93–50.62)	0.962	0.0915	0.4417	
10 years or above	9	509	243	51.51 (34.52–68.34)	0.932	< 0.001	0.1705	
**By province**								0.0573
Punjab	11	2113	1014	45.98 (38.15–53.90)	0.923	< 0.001	0.1496	
Sindh	6	857	236	31.81 (20.27–44.59)	0.928	< 0.001	0.0922	
Khyber Pakhtunkhwa	8	1386	362	28.04 (13.58–45.26)	0.976	< 0.001	0.3754	
**By period**								0.5388
1995–2004	6	708	223	41.27 (28.15–55.03)	0.913	< 0.0001	0.0050	
2005–2014	12	2017	822	38.03 (28.13–48.45)	0.956	< 0.001	0.2211	
2015–2018	9	3064	902	30.66 (18.06–44.91)	0.983	< 0.001	0.5755

The results of the univariate meta-regression analysis of the prevalence of HCV in *β*-thalassemia patients are presented in Table 3. The analysis shows that only geographical region (province) had a significant effect on the prevalence of HCV in *β*-thalassemia patients with a *p*-value < 0.1, while year of publication, year of study, sample size, proportion of males, average age of thalassemia patients had no significant effect on the observed HCV prevalence in *β*-thalassemia patients.

**Table 3:** Results of bivariate meta-regression for prevalence of HCV infection in *β*-thalassemia patients in Pakistan

Covariate	Category	Number of Studies	Meta-regression Coefficient (%)	*p*-value	Variance explained *R*^2^ (%)
Geographical region (Province)	Khyber Pakhtunkhwa	8	1		26.97
	Punjab	11	0.1873	0.0306	
	Sindh	6	0.0447	0.6582	
Year of publication		27	−0.0098	0.1176	1.33
Year of study		21	−0.0076	0.3058	0.00
Sample size	< 100	8	1		2.92
	> = 100	19	−0.1329	0.1187	
Proportion of males		23	−0.0003	0.9189	0.00
Average age of patients		25	− 0.0058	0.6598	3.41
Sample size, continuous		27	−0.0098	0.1176	1.33

## Discussion

The aim of this study was to summarise the available literature on the prevalence of hepatitis C virus infection in *β*-thalassemia patients and its correlated risk factors in Pakistan. The result of this meta-analysis showed that the pooled prevalence based on 27 studies was 36.21%. More than one in every three *β*-thalassemia patients in Pakistan have already been exposed to HCV infection. The pooled prevalence of HCV in *β*-thalassemia patients, as showed by this study is six times higher (36.21%) than in the general Pakistani population which is 6.2% [45]. In Pakistan, many patients with *β*-thalassemia have limited access to regular and safe blood transfusions. Possible reasons for this are the lack of altruistic voluntary blood donors and the inadequate testing of blood donations for HCV. Many blood transfusion centers and hospitals have inadequate resources and kits for screening blood donations [5]. The root cause of the high prevalence is predominantly the lack of adequate regulation of blood banks and monitoring to assess compliance with transfusion safety standards. It is well recognized that, with proper regulation driven by policy makers, transfusion transmitted infections are markedly reduced [5]. Pakistan is a low resource country: the pooled prevalence of HCV in *β*-thalassemia patients in Pakistan is higher than that in Iran [46] (19%) or Bangladesh [47] (14.7%). The findings of this study should act as a major safety alert for decision and policy-makers in the Pakistani health sector.

Our data on HCV infection prevalence among the *β*-thalassemia patients covers all provinces of Pakistan except Baluchistan and Gilgit-Baltistan. Our results showed that the prevalence of HCV infection in *β*-thalassemia patients was higher in Punjab (45.98%) than in Sindh (31.81%) and Khyber Pakhtunkhwa (28.04%).

In this paper, we observed that the prevalence of HCV in *β*-thalassemia patients rises with age, increasing from 33.87% in the under 10 years age group to 51.51% in the 10 years or above age group. This effect was not statistically significant at conventional levels. We believe that age is acting as a proxy for other effects. Age is associated with cumulative exposure to blood transfusions over a life time and it is the number of blood transfusions which is associated with increased risk of HCV infection. Unfortunately, we do not have data on the number of blood transfusion patients had received. Conversely, one could look at this more positively and suggest that the frequency of testing for HCV positive blood donations has improved and hence younger patients have a lower infection rate than their older fellow patients did when they were the same age, due to safer blood donations.

Meta-regression analyses showed that there was no significant change in the prevalence of HCV in *β*-thalassemia patients over the past three decades (with both years of publication and year of study (data collection).

To our knowledge, this is the first systematic review and meta-analysis to compile current data on the prevalence of HCV infection among *β*-thalassemia patients in Pakistan. The main strengths of this study are the use of a comprehensive and a predefined literature search strategy, and the involvement of two independent reviewers in the whole review process and data extraction. No publication bias was found within our analyses which suggests that we are unlikely to have missed any potential studies that could change the results of this meta-analysis. Furthermore, the methodological quality of all included articles had a low risk of bias. As showed by meta-regression analysis, the methodological quality of the studies had no influence on pooled prevalence estimates. Three provinces of Pakistan were covered in the investigation of HCV infection prevalence in *β*-thalassemia patients. On the other hand, the findings of this study have some limitations. Firstly, the meta-regression analysis was only based on bivariate analysis. We planned to use a multivariate meta-regression model by considering all the factors simultaneously, however, it was not possible to use multivariate meta-regression analysis due to the small number of studies. A multivariate meta-regression analysis requires at least ten studies per factor to estimate the meta-regression coefficients efficiently. Second, and as is common in meta-analyses, the study estimates revealed substantial heterogeneity between the included studies, which may be due to the other sources of variation may have been missed in our analysis, such as the number of blood transfusions, some genetic factors, and type of *β*-thalassemia; but we were unable to investigate these factors due to lack of data.

## Conclusions

The overall prevalence of HCV in *β*-thalassemia patients in Pakistan was 36.21%, but varied from province to province. The prevalence is higher than in neighboring countries such as Iran and Bangladesh. Pakistan is a developing country and lacking in resources for appropriate blood screening facilities in thalassemia centers and hospitals. Lack of robust policies on transfusion safety as well as appropriate and rigorous monitoring of blood banks to ensure compliance with policies perpetuate the risk of transfusion transmitted infection with HCV. National and regional health programs should mandate and monitor the screening procedures so as to reduce the risk of transfusion transmitted infections such as HCV in the general population in *β*-thalassemia patients.

## Data Availability

All relevant data is included within the manuscript file.

## Abbreviations

CI: 95% confidence interval
ELISA: Enzyme-Linked Immunosorbent Assay
HCV: Hepatitis C virus
PRISMA: Preferred Reporting Items for Systematic Reviews and Meta-Analyses
RNA: Ribonucleic Acid
